# Post-traumatic stress disorder among ICU healthcare professionals before and after the Covid-19 health crisis: a narrative review

**DOI:** 10.1186/s13613-023-01145-6

**Published:** 2023-07-21

**Authors:** Victoire Deltour, Anne-Laure Poujol, Alexandra Laurent

**Affiliations:** 1 Psychology Laboratory: Relational Dynamics and Identity Processes (Psy-DREPI), University of Bourgogne Franche-Comté, AAFE pole, Esplanade Erasme, 21078 Dijon, France; 2 Multidisciplinary Intensive Care Unit, Department of Anesthesiology and Critical Care, La Pitié-Salpêtrière Hospital, Assistance Publique-Hôpitaux de Paris, Sorbonne University, Paris, France; 3 VCR Team, School of Practitioner Psychologists, Catholic University of Paris, 7403 Paris, EA France; 4 Department of Anaesthesiology and Critical Care Medicine, Dijon University Medical Centre, Dijon, France

**Keywords:** Narrative review, PTSD, Intensive care unit (ICU), Healthcare professionals, Covid-19

## Abstract

**Background:**

The ICU (intensive care unit) involves potentially traumatic work for the professionals who work there. This narrative review seeks to identify the prevalence of post-traumatic stress disorder (PTSD) among ICU professionals; how PTSD has been assessed; the risk factors associated with PTSD; and the psychological support proposed.

**Methods:**

Three databases and editorial portals were used to identify full-text articles published in English between 2009 and 2022 using the PRISMA method.

**Results:**

Among the 914 articles obtained, 19 studies met our inclusion criteria. These were undertaken primarily during the Covid-19 period (*n* = 12) and focused on nurses and assistant nurses (*n* = 10); nurses and physicians (*n* = 8); or physicians only (*n* = 1). The presence of mild to severe PTSD among professionals ranged from 3.3 to 24% before the pandemic, to 16–73.3% after the pandemic. PTSD in ICU professionals seems specific with particularly intense intrusion symptoms. ICU professionals are confronted risk factors for PTSD: confrontation with death, unpredictability and uncertainty of care, and insecurity related to the crisis COVID-19. The studies show that improved communication, feeling protected and supported within the service, and having sufficient human and material resources seem to protect healthcare professionals from PTSD. However, they also reveal that ICU professionals find it difficult to ask for help.

**Conclusion:**

ICU professionals are particularly at risk of developing PTSD, especially since the Covid-19 health crisis. There seems to be an urgent need to develop prevention and support policies for professionals.

## Introduction

The ICU receives, within a context of emergency and unpredictability, patients with serious pathological conditions whose vital risk is engaged. In these services, the mortality rate is high, varying between 20 and 25% depending on the services, and the threat of patient death is omnipresent [1]. The announcement of difficult diagnoses and severe prognoses is part of the daily life of professionals, to which must be added the patients’ moral and physical suffering, the degradation of the body, and the pain of families experiencing care of a loved one who is in distress and fear.

Since 2020, there have been unprecedented epidemic waves characterized by extreme tension [2] related to the risk of being contaminated or of contaminating others, and to the lack of trained intensive care personnel and of personal protective equipment for these workers. In addition, the lack of specific treatments, patient deaths, and the distress of families deprived of visits also contributed to a sense of helplessness among caregivers. Finally, issues of bed availability and the risk of patient triage raised many ethical and moral dilemmas within the ICU teams [3].

This work environment brings together all the risk factors for PTSD described by the Diagnostic Manual of Mental Disorders (DSMV) [4]: having to confront death—and the threat of death or serious illness—personally, or having to deal with a loved one’s (family member’s or close friend’s) serious illness. The clinical picture of PTSD is dominated by three dimensions, 1/the intrusion symptoms with nightmares, repetitive memories, hallucinations, or heightened states of alertness about traumatic event; 2/avoidance symptoms in an attempt to avoid thinking about, or having to face, traumatic situations; and 3/neurovegetative symptoms such as sleep disorders, hypervigilance, and irritability [4]. Shame and guilt are also widely described and are frequently associated with depressive manifestations related to feeling at fault [5]. All these symptoms are observable more than 1 month after exposure to the traumatic event.

We aimed to undertake a scoping review of published literature concerning PTSD in ICU healthcare workers. We focused on three broad questions: first, what is the prevalence of PTSD in critical care workers in published research before and after COVID?; second, what are the specificities of PTSD in intensive care workers and the risk factors associated with PTSD?; third, what are protective factors and the psychological support proposed by studies ?

## Material and methods

This literature review is based on the narrative review method, which is preferred when one wishes to conduct an analysis of a set of studies that have used diverse methodologies with different theoretical conceptualizations [6]. Narrative reviews synthesize the results of studies without reference to the statistical significance of the results [7]. They are relevant when one wishes to make connections between studies in order to develop or evaluate a new theory or future recommendation.

### Databases and search strategy

We considered all peer-reviewed full-text articles reporting an empirical study published in English between 2009 and 2022. The search for, and selection of, articles were conducted between January and March 2022, according to PRISMA guidelines. Five databases and editorial portals (Medline via PubMed, PsychINFO, Web of Science, Elsevier’s ScienceDirect, and Ovid (APA) were screened using the following terms in the title, the abstract, the keywords or the MeSH headings: (“Post-traumatic stress disorders” OR “PTSD” OR “Traumatic stress” OR “Acute stress disorder”) AND (“Healthcare” OR “Healthcare workers” OR “healthcare providers” OR “physicians ICU” OR “Nurse”) AND (“Intensive care units” OR “ICU” OR “Critical care”). We had no limitations based on geography, healthcare systems, or demographic factors. This choice of keywords allowed us to bring out the studies concerning ICU and PTSD with all the professionals combined.

### Eligibility criteria

Quantitative, qualitative, and mixed-methodology studies examining PTSD in ICU healthcare workers (HCW) were included. Studies had to be conducted on ICU nurses, assistant nurses, physicians, and residents working with adult patients.

We excluded the following studies: (1) systematic; meta-analysis; empirical research; feedback; letters to the editor; recommendations; (2) studies focused on a population other than the ICU professionals included in our study (physical therapists; radiologists; administrative services; and so on); (3) studies conducted among sectors other than adult patients in ICU, such as patients within pediatric ICU or emergency services; (4) studies whose diagnosis did not relate to direct exposure to trauma, e.g., secondary or vicarious trauma or symptomatology assessed within 1 month of the event, e.g., acute stress or traumatic dissociation; (5) studies that addressed disorders other than PTSD, e.g., compassion fatigue, anxiety, burnout; (6) studies that addressed resiliency without examining trauma; (8) studies that spoke of trauma as a possible outcome but which did not measure it.

### Selection process and data extraction

**Fig. 1:**
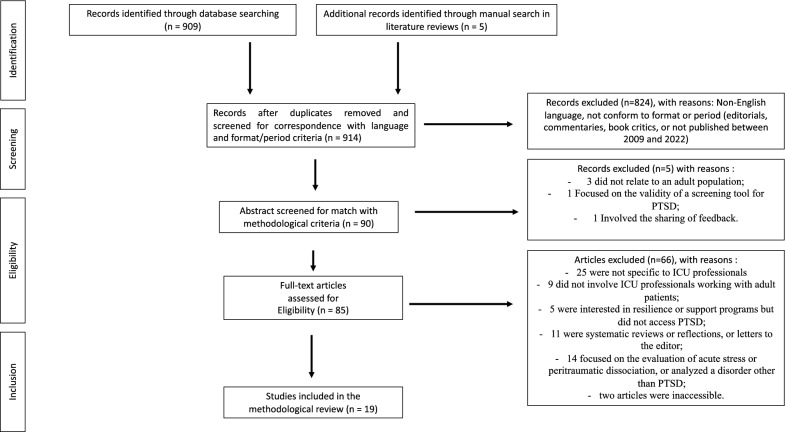
Flow-diagram of selected articles

The results were screened by title and abstract by three independent reviewers (VD, AL, ALP). Where reviewers were in disagreement, the reasons for this were discussed and consensus was reached. Relevant articles were retrieved for full-text reading to identify relevant studies. Each reviewer (VD, AL, ALP) independently reported the article data in an Excel table in order to justify clearly the selection or exclusion of an article for the other reviewers. Data collected were author, title, year of publication, research country, aim of the research, method, population, and main results. In addition, when the data were clarified, reviewers noted the prevalence of PTSD in the population and the PTSD symptoms, as well as factors of vulnerability, protective factors, and psychological mechanisms for supporting HCW with trauma. Outcomes from the search terms and screening were summarized using a PRISMA flow-diagram (Fig. 1). As this was a scoping review, no formal quality analysis or summary statistics were undertaken.

## Results

A narrative review of studies focusing on PTSD in ICU HCW for adult patients led to the identification of 914 potentially relevant articles (of which three studies were identified manually and added as additional references) (Fig. 1). After removing duplicates and screened for correspondence with language and format/period criteria (*n* = 824), the abstracts of 90 articles were reviewed. A total of five articles were eliminated from the outset, either because they did not relate to an adult population (*n* = 3), they focused on the validity of a screening tool for trauma (*n* = 1), or they involved the sharing of feedback (*n* = 1). Of the remaining 85 articles, 25 were not specific to ICU professionals (e.g., frontline healthcare professionals involving all medical specialties); nine did not involve ICU professionals working with adult patients; five were interested in resilience or support programs but did not access PTSD; 11 were systematic reviews or reflections, or letters to the editor from authors on the topic; 14 focused on the evaluation of acute stress or peritraumatic dissociation that had occurred within a month of the event, or analyzed a disorder other than PTSD (e.g., burnout, anxiety, depression); and two articles were inaccessible.

Overall, 19 studies were included in this narrative review. These mainly concern: nurses and assistant nurses (*n* = 10); nurses and physicians (*n* = 8); or physicians only (*n* = 1). They were principally conducted during the Covid-19 period (*n* = 12), in France (*n* = 3), in Canada (*n* = 2), in the Netherlands (*n* = 1), in Italy (*n* = 2), in the United Kingdom (*n* = 1), in the United States (*n* = 2), and in Belgium (*n* = 1). Only seven studies conducted before the Covid-19 pandemic were included; these were conducted in South Korea (*n* = 1), the Czech Republic (*n* = 1), the United Kingdom (*n* = 1), the United States (*n* = 3), and Australia (*n* = 1) (Table 1).

**Table 1:** Articles identified after a narrative review according to the research country, the period (before or during the Covid-19 pandemic), the study method, the population of ICU healthcare workers included, the PTSD measurement tool, the risk and protective factors for PTSD in this population, the prevalence of PTSD, and the proposed psychological support

Author (date) ^ref^ Country	Purpose of study *Extracted from the summary of the article*	Study design	Tools (for PTSD*)	Sample HCW = health care workers	PTSD results (risk factors, protective factors, PTSD support …)
Before the pandemic					
Cho, et Kang [37] South Korea	“To investigate the relationship between Type D personality and post-traumatic stress disorder (PTSD) symptoms in ICU nurses.” “To determine the mediating effect of resilience on this relationship.”	A prospective, cross-sectional, multicentric study Quantitative method	DS 14 Post-traumatic Diagnostic Scale (PDS)* CD-RISC	179 nurses	18.2% of ICU professionals with type D personality were considered to be at a higher risk of developing PTSD A type D personality is positively correlated with PTSD and negatively correlated with resilience PTSD Support: Early assessment of PTSD in healthcare professionals, building resilience
Colville, et al. [38] UK	“To examine the associations with symptoms of 1) burnout and 2) work-related post-traumatic stress in adult and pediatric intensive care staff, focusing on the particular contributions of resilience and coping strategies.”	A prospective, cross-sectional, multicentric study Quantitative method	BRS aMBI Trauma Screening Questionnaire (TSQ)* HADS List of coping strategies List of options for additional forms of support	377 HCW	52% reported between one and five symptoms in the previous fortnight, and 13% reported six or more symptoms, indicating that they were potentially at risk of PTSD because of an experience they had at work and that they might benefit from further psychological assessment Risk factors associated with PTSD: Suffering from burnout; working in pediatric ICU; critical care nurses with a graduate degree in nursing Protective factors: Among the coping strategies, seeking of social support is particularly used but is associated with greater distress; resilience; outdoor activities (leisure); discussions with "senior citizens"; expression of emotions PTSD Support: More debriefing; more time for reflective practice; training in mindfulness or relaxation; spending more time with their managers and having more training, especially on the range of psychological reactions that they may experience, to help them to identify and address symptoms earlier
Dennis, et al. [27] Australia	“To elicit the nature and sources of workplace emotional distress in an international sample of intensivists.”	A retrospective, cross-sectional, multicentric study Qualitative method	Semi-structured interview	19 physicians	Risk factors: Experiencing clinical situations that remind one of past traumatic situations PTSD Support: Explicit training in negotiation, difficult conversations, effective supervision; consensus building, which seems to be common in the business and leadership literature, seem very relevant for Intensivists
Janda, et al. [8] Czech Republic	“To determine the occurrence of post-traumatic symptoms and symptoms of anxiety and depression among nurses in a Czech tertiary hospital.” “To explore the relationship between stressful work factors, work overload, and disturbed interpersonal relationships, and the appearance of their symptoms.”	A prospective, cross-sectional, multicentric study Quantitative method	PTSS-10* HADS	151 nurses	3.3% of ICU nurses have signs of PTSD but there is no information on the duration between the traumatic event and the moment when the symptoms are assessed Risk factors: Caring for an organ donor; insufficient nursing staff
McMeekin, et al. [9] USA	“To explore the relationships between postcode stress, coping behaviors, and PTSD symptom severity in critical care nurses after experiencing unsuccessful cardiopulmonary resuscitations, and to see whether institutional support attenuates these repeated psychological traumas.”	A retrospective, cross-sectional, multicentric study Quantitative method	PCSS Brief cope The Impact of Event Scale (IES-R)*	490 nurses	23.7% of nurses have a high score at four months of resuscitation failure Risk factors associated with PTSD: The study identifies being female and having so-called “ineffective” coping strategies (denial, self-distraction, self-blaming, behavioral disengagement); debriefings; or participation in ICU failures Protective factors: Active adaptation, instrumental support, acceptance
Mealer, et al. [39] USA	“To determine if a multimodal resilience training program for ICU nurses was feasible to perform and acceptable to the study participants.”	A single-center, prospective, randomized, and controlled study Quantitative method	12 weeks intervention CD-RISC The Post Traumatic Diagnostic Scale (PDS)* HADS MBI CSQ-8	27 nurses	44% of ICU nurses have signs of PTSD Resilience is the protective factor studied primarily in this study PTSD Support: Develop resilience among ICU nurses by proposing adapted programs
Mealer, et al. [19] USA	“ To determine whether post-traumatic stress disorder (PTSD) and burnout syndrome (BOS) are common in nurses, and whether the co-existence of PTSD and BOS is associated with altered perceptions of work and non-work-related activities”	A single-center, prospective randomized, and controlled study Quantitative method	HADS PTSS-10* PDS MBI	332 nurses	11% of ICU nurses have a positive score on the PTSD diagnostic scale 33% of ICU nurses have signs of PTSD Risk factors associated with PTSD: The study identifies be exposed to a traumatic event such as witnessing patients dying, massive bleeding, open surgical wounds, trauma-related injuries, and providing futile care to critically or terminally ill patient Age (younger people) and year of experience (less you had experience more chance you had to be trauma) was also associated with PTSD
During the pandemic					
Altmayer, et al. [10] France	“To investigate and to compare the psychological impact of the pandemic on ICU regular staff and on reinforcement.”	A prospective, cross-sectional, single-center study Quantitative method	HADS The Post-traumatic Stress Disorder Checklist (PSDC)* MQOL-R CD-RISC-10	69 HCW	16% of HCW have PTSD, assessed at the peak of the first wave (online survey available from March to April 2020) Risk factors: Team reorganizations during the first wave (reception and training of reinforcement personnel); lack of recognition of the work done; taking responsibility for errors committed by the reinforcement personnel The protective factors identified are the sense of belonging, the time spent in intensive care, and the age of the healthcare professional PTSD Support: free and easy access to a psychologist during such a period
Caillet, et al. [12] France	”To assess the psychological impact of Covid-19 on the healthcare professionals at the peak of the ‘crisis period’.”	A prospective, cross-sectional, multicentric study Quantitative method	HADS IES-R*	208 HCW	27% of participants, at the acute phase of the first peak (April 8 to 21, 2020) Protective factors: Informing ICU professionals about the Covid-19 outbreak (mode of transmission, prevention procedure) could reduce the associated stress PTSD Support: Managers must be vigilant when dealing with professionals prone to psychological disorders. In addition, training and/or reorientation should be considered to prevent psychological repercussions on teams during a new health crisis or a traumatic event
Carmassi, et al. [16] Italy	”To investigate post-traumatic stress symptoms (PTSS), anxiety and depressive symptoms, and their relationship with impairment in the functioning impairment among frontline HCWs from three Italian regions differently exposed to the first wave of the Covid-19 emergency: Tuscany (low), Emilia-Romagna (medium), and Lombardy (high).”	A prospective, cross-sectional, multicentric study Quantitative method	IES-R* PHQ-9 GAD-7 WSAS	129 ICU HCW / 514 HCW	IES-R score 18.79 ± 21.02, p < 0.05, during the acute phase of the first peak of the Italian outbreak Risk factors: Being a nurse PTSD Support: Enable early detection of PTSD and facilitate access to psychiatric care following a pandemic
Crowe, et al. [11] Canada	”To examine the mental health of CCRNs providing direct patient care during the initial phase of the Covid-19 pandemic in Canada.”	A retrospective, cross-sectional, single-center study Mixed method	Semi-structured interviews IES-R* DASS-21	109 nurses	73.3% of critical care professionals show signs of PTSD two months before the onset of the crisis (May 2020) Risk factors: Fear and anxiety associated with the virus and with contamination; poor communication; substantial and unclear information PTSD Support: Organization of stress management sessions; regular monitoring of healthcare professionals with the intervention of a psychiatrist
Foli, et al. [20] USA	“To describe the experiences of frontline nurses who are working in critical care areas during the Covid-19 pandemic with a focus on trauma and on the use of substances as a coping mechanism.”	A retrospective, cross-sectional multicentric study Qualitative method	Two open-ended items	73 nurses	One month before the beginning of the crisis (mid-June 2020 to early September 2020), nurses speak of psychological distress that takes various forms. This is marked by having to face frequent death and end-of-life situations, and by conflict that arises between the fear of being contaminated and of contaminating one's loved ones, on the one hand, and undertaking one's professional duties on the other hand; a sense of institutional and societal betrayal related to constant changing of practice guidelines and public ignorance, perceived lack of support, insufficient resources (personal protective equipment, resources for patient care) PTSD Support: Provide organizational and governmental support for healthcare professionals. Also provide individual support in the workplace
Greenberg, et al. [13] UK	“To identify the rates of probable mental health disorder in staff working in ICUs in nine English hospitals during June and July 2020.”	A prospective, cross-sectional, multicentric study Quantitative Method	GAD PHQ-9 The 6-item Post-traumatic Stress Disorder checklist (PCL-6)* AUDIT-C WEMWBS	709 HCW	45% of the sample reporting probable PTSD symptoms, severe depression, or severe anxiety disorder at three months of the fifth peak (June to July 2020) Risk factor: Being a nurse PTSD Support: Become conscious of the pathological risk incurred by professionals for preventive and therapeutic purposes; provide timely access to treatment for the professionals who need it; ensure the support of supervisors and peers
Heesakkers, et al. [21] Netherlands	“To determine the impact of the first Covid-19 surge (March through June 2020) on mental well-being and the associated risk factors among intensive care unit nurses.”	A prospective, cross-sectional, multicentric study Quantitative method	HADS-A HADS-D IES-6* NFR	726 nurses	22.2% of the sample showing signs of PTSD more than 1 month before the first wave (online study from August to September 2020) Risk factors: Working in a University Hospital Center, fear of contaminating relatives, insufficient staff, working longer hours or doing longer shifts PTSD Support: Optimize working conditions, reduce workload, ensure a sufficiently large and trained team
Hernandez, et al. [33] USA	“To assess the prevalence of traumatic stress among American frontline nurses following the initial Covid-19 surge in the United States during March 2020, using the Trauma Screening Questionnaire.”	A prospective, cross-sectional, multicentric study Quantitative method	The Trauma Screening Questionnaire (TSQ)*	298 nurses	One month after the beginning of the crisis, 58.7% are suffering from PTSD with disturbing thoughts or memories of the event. They feel upset when they remember the event; they have sleep disorders and an increased awareness of dangers Risk factors: Being a nurse; being female; an increased incidence of Covid-19; lack of personal protection and other medical equipment; insufficient knowledge about the virus PTSD Support: Educate nurses about their mental health; early detection of PTSD; train clinical psychologists to support frontline professionals in such situations; develop research on PTSD
Lasalvia, et al. [14] Italy	”To assess the magnitude of psychological distress and associated factors among hospital staff during the Covid-19 pandemic in a large tertiary hospital located in north-east Italy.”	A prospective, cross-sectional, single-center study Quantitative method	IES-R* SAS PHQ-9	2195 HCW	66% of HCW reported symptoms of PTSD during the first wave (21 April to 6 May 2020) Risk factors: Being female; being a nurse; working in a Covid-19 unit or in an ICU; living alone; having more than 20 years’ professional experience; having a psychopathological history; fear of being infected with Covid-19; demanding working conditions; having to deal with death and end-of-life situations PTSD Support: - Active monitoring of reactions and performance, altering assignments and schedules, modifying expectations, assessing occupational risks, and offering − where necessary − psychosocial support - Adapting the type of intervention to the time of the crisis and the specific needs − ranging from peer support to professional aid - To prevent the development of psychological disorders in the long term, active monitoring and provision of psychological support should be delivered once the crisis begins to recede (Greenberg et al., 2020) - Develop research on the long-term effects of the pandemic, and on proposed interventions to support healthcare professionals' mental health
Laurent, et al. [15] France	”To measure the prevalence of post-traumatic stress disorder in HCWs.” “To identify risk factors and protective factors during the epidemic in France.”	A prospective, longitudinal, multicentric study Mixed method	PS-ICU scale GHQ 12 Brief cope IES-R* Open questions	2153 HCW	Three months after the beginning of the first peak (June-July 2020), 24.10% of doctors, 29.68% of residents, 32.78% of nurses, 32.80% of medical students, 34.85% of nurses, and 36.36% of caregivers show signs of PTSD Risk factors: Being female; experiencing other difficult events during the crisis; having a high score of psychological distress during the crisis; having a high level of perceived stress related to workload and human resource issues; an emotional load related to the patient and family; and having a high level of perceived stress related to the risks of contamination, powerlessness, and insecurity associated with the crisis Protective factors: Turning to positive thinking PTSD Support: Set up support systems adapted to the crisis situation for professionals, and allow them to develop positive-thinking coping skills
Van Steenkiste, et al. [40] Belgium	“To assess the mental health impact of Covid-19 on nurses working on the frontline during the first wave of Covid-19 hospitalizations in Belgium in 2020.”	A prospective, longitudinal, single-center study Quantitative method	4 DSQ IES-R* Brief cope	39 nurses	10% of participants were at risk of PTSD during the first peak (1 April to 30 June 2020) Risk factors: High workload, poor communication, caring for Covid-19 patients PTSD Support: Use a multidisciplinary support team (psychologists, palliative support team, philosophical services, social workers, liaison psychiatrist, and palliative doctors) to increase resilience by means of individual sessions where needed; discuss themes such as “dealing with change in times of crisis”, “preservation of, resilience”, “work/life balance”, and “burnout prevention” on a regular basis; develop external training and coaching modules for head nurses and an internal e-learning module
Mehta et al. [17] Canada	“To evaluate the impact of the COVID-19 pandemic on Canadian intensive care unit (ICU) workers”	A prospective, cross-sectional multicentric study Quantitative method	IES-R* K-10	455 ICU’S HCW	37% have signs of PTSD 25% have an IES-R score indicating a probable diagnosis of PTSD 33% of nurse had an IES-R score indicating a probable diagnosis of PTSD VS 5% of physicians Nurses have a higher score of IES-R than physicians Risk factors: female sex, high-risk health status, living with a child or children, and feeling at increased risk because of PPE shortage or inadequate training

*Scales assessing PTSD in the study

### High prevalence of PTSD evaluated among ICU professionals in the wake of the Covid-19 health crisis

Our literature review of PTSD in ICU professionals working with an adult population over the 10 years preceding the pandemic (2009–2019), as well as during the pandemic (2020–2022), shows a renewed interest in the question of PTSD in healthcare professionals. Indeed, while our analysis over a 10 year period revealed seven studies before the crisis, 12 studies were undertaken during the Covid-19 period. The increase in the number of studies goes hand in hand with an increased risk of PTSD during this pandemic period. Indeed, studies conducted before the Covid period show the prevalence of PTSD in ICU professionals ranging from 3.3% [8] to almost 24% [9]. During the Covid period, 16% [10] to 73.3% [11] of professionals have mild to severe PTSD. While all socio-professional categories are affected [12–15], nurses are the most vulnerable to PTSD [12–17]. Finally, the IES-R [18] seems to be the measure used most often to assess PTSD in ICU professionals. This is a scale based on the DSM-V criteria [4] with three major dimensions: intrusion, avoidance, and neurovegetative symptoms.

### The characteristics of PTSD in ICU healthcare professionals and risk factors

Regarding the symptomatology of PTSD in ICU professionals, intrusion symptoms appeared to be one of the most significant disorders [14]. Studies show that healthcare professionals in intensive care are confronted with risk factors for PTSD, the most important of which are related to death (death of patients, end-of-life, organ removal) [8, 19]. During the Covid crisis, the experiences of confronting death and seriously ill patients were intensified by the violence and duration of the crisis [20]. In this sense, new factors emerged that were associated with the unpredictability and uncertainty of healthcare, and with the insecurity linked to the risk of being contaminated, or of contaminating one’s family, with Covid-19, thus affecting HCW professionally and personally [11, 12, 14, 15, 17, 20, 21].

### Protective factors and support systems recommended in the studies identified

Few healthcare professionals took advantage of the psychological supports available during the crisis, such as hotlines [10, 15, 22]. Laurent and her colleagues [15] noted that professionals turned primarily to more local forms of support, such as relatives, colleagues, the hierarchy, and ICU psychologists. It appears that the development of strategies based on positive thinking within ICUs had a protective effect against PTSD [15].

All the studies analyzed emphasized the importance of clear and smooth communication during a crisis in order to limit—as far as possible—feelings of fear and helplessness [11, 21]. Moreover, feeling as if one were safe in one’s own department, one had one’s team’s support, and also had sufficient material and human resources, seemed to be protective factors against PTSD [16, 21].

## Discussion

Our literature review shows that it seems essential to pay particular attention to the risk of trauma in intensive care teams. Indeed, following the crisis, the prevalence of PTSD has increased to 73.3% among intensive care professionals. More than 1 year after the COVID crisis began, the prevalence of the disorder remains high (13, 7% [23] and 37% [24]). This prevalence of PTSD among ICU caregivers appears in the majority of studies in our review to exceed the prevalence of the population of health professionals, all specialties combined during the COVID crisis [25] and of the general population, which, outside the COVID period, is 8.3% over a lifetime in US [26], and more specifically in France in 2008 at 0.7% with an almost equal frequency between men and women [27].

We need to understand the traumatic risk, and in particular intrusion symptom, in the ICU in relation to the work context of the professionals. Firstly, experiencing a traumatic event in the context of work does not facilitate either the possibility of psychological reconstruction or the reduction of symptoms. Indeed, it should notice that HCW continued to work in the same environment that had caused the trauma and therefore could be impacted again, at any time, by an event that could rekindle the traumatic situation previously experienced within the service (e.g., same pathology, same room, same team). Traumatic events could be stored in one’s memory for a long time and thus be more likely to generate strong emotional reactions when HCW are faced with a similar situation [28].

The importance of PTSD in the intensive care population also leads us to question the link with other psychological difficulties highlighted in this population, notably burnout [29]. The literature highlights the link between PTSD and burnout [30], particularly during the health crisis which led to deteriorated working conditions and loss of confidence in an institution and a healthcare system that have been unable, or hardly able, to protect their workers [31]. This context makes professionals even more vulnerable, as shown in the Italian study by Lasalvia et al. [14], during the Covid period, professionals who had experienced a traumatic event at work related to Covid-19, more frequently obtained scores above the cut-off point on the Maslach Burnout Inventory (MBI). Both at the beginning of the pandemic and two months after the crisis, studies show that the higher the level of burnout, the more severe the post-traumatic stress symptoms [32, 33]. Therefore, according to Lui et al., any initiative that helps to alleviate burnout may be useful in preventing the severity of PTSD [33].

We would like to draw attention to the fact that the results of our narrative review should also be discussed with regard to the methodology on which the identified studies are based. While the DSM-V criteria [4] relate to the international level, there is nevertheless some nosographic confusion within the studies between—on the one hand—what may be described as acute stress in the month following the event and—on the other hand—PTSD from which the healthcare worker suffers chronically: only five studies out of 17 clearly explained the temporality of the assessment of disorders. However, as Hernandez and her colleagues [34] state in their study, to diagnose PTSD effectively, one must consider the intensity of the event, on the one hand, and—on the other—the psychotraumatic manifestations arising from. Indeed, the intensity of the event and its immediate impact are not sufficient for diagnosing PTSD, it is the long-term condition of the disorders—beyond a month—that reveals the traumatic scope of the event [4].

Finally, few studies have really investigated and evaluated the relevance of prevention or support measures. It should be noted that the inability of the HCW staff to ask for help remained a significant problem. Indeed, although the available studies highlight the suffering of HCW [10], it appears that initiating a process of help or care is still difficult for healthcare professionals [15]. This results primarily from the emergency context which is associated with time constraints, heavy workloads, ignorance of psychologists’ work [15], and a medical culture of uncomplaining healthcare professionals, [10] and which does not make this process any easier. In view of the high risk of PTSD in the intensive care population, it seems essential to develop more interventional studies to measure the relevance of psychological devices within the wards. In parallel, it seems necessary to set up psycho-educational seminars for caregivers to better understand the risks of trauma, as this is a first step to enable caregivers to better identify risk situations and their consequences on their mental health [35]

This study has several limitations. Some studies may have been overlooked if their article titles, keywords, or abstracts did not clearly reflect the objective of identifying PTSD in ICU healthcare professionals. In addition, studies developed at a more local level may have been excluded from our review. Given that meta-analysis was not feasible or appropriate because of the nature and design of the studies, a narrative approach was used to summarize the data, as recommended for scoping reviews. Lastly, the Covid-19 health crisis is recent and it is still difficult to account for the real traumatic impact of this crisis on healthcare professionals over time.

## Conclusion

ICU professionals are exposed to traumatic situations that can have a long-term impact and it is essential today to set up prevention and psychological support measures for this population. Studies on measures to help professionals to cope with trauma are rare. These are also contradictory because, while they advocate easier access to psychological care for ICU professionals and the active participation of these professionals and the hospital system, they underscore how difficult it is for professionals to ask for help, and for the hospital system to find resources to provide appropriate care [36].

The health crisis seamlessly transitioned from an acute and exceptional phase to a chronic phase taking the form of a normality at work [15]. The risk is to have transformed the ICU into a space of repetitive trauma without the professionals having been able to debrief them.

## Data Availability

No concerned.
